# Editorial: Extrapulmonary manifestations of SARS-CoV-2 infection and COVID-19 vaccine adverse effects

**DOI:** 10.3389/fcimb.2025.1688071

**Published:** 2025-09-09

**Authors:** Monica Catarina Botelho, Anello Marcello Poma, Jian Wu

**Affiliations:** ^1^ Instituto de Investigação e Inovação em Saúde, Universidade do Porto, Porto, Portugal; ^2^ Instituto Nacional de Saúde Dr Ricardo Jorge, Porto, Portugal; ^3^ Department of Surgical, Medical, Molecular Pathology and Critical Area, University of Pisa, Pisa, Italy; ^4^ Suzhou Municipal Hospital, Suzhou, China

**Keywords:** SARS- CoV- 2, long COVID, extrapulmonary COVID, autoimmune diseases, vaccine (COVID-19), tumor growth

It is well-established that COVID-19 is a systemic disease. Besides the respiratory system, the virus can infect different extrapulmonary tissues, and this infection can determine histopathological and molecular changes that disrupt the normal functions of the organism. Neurologic disorders, abnormal glycemic control, gastrointestinal symptoms, and even endocrine dysfunctions belong to the spectrum of COVID-19. Although these symptoms can be limited to the acute phase of infection, a significant number of patients with COVID-19 experience prolonged sequelae, even for weeks or months. The scientific community and general public are already accustomed to the terminology “long COVID” which is used to define this post-acute disease manifestation, which mirrors the late consequences of the viral insult.

The Virus and Host section of the journal Frontiers in Cellular and Infection Microbiology, hosted the Research Topic on Extrapulmonary manifestations of SARS-CoV-2 infection and COVID-19 vaccine adverse effects with the aim to facilitate global COVID-19 elimination through scientific advances.

More than 50 authors, representatives from China, Romania and Spain participated in this Research Topic.

This Research Topic comprises six papers, spanning topics that cover diagnostic markers, epidemiology, mechanisms of virus-host association, potential therapeutics, host pathology, virus biology, health burden, prevention and control programs, and public health policy.

Chun Yan He from Kunshan Hospital of Chinese Medicine, Affiliated Hospital of Yangzhou University (Kunshan, China) presented a work on serum hyaluronic acid and procollagen III, N-terminal propeptide levels as biomarkers of disease severity and progression prediction of COVID-19 (Yang et al.).

Hua Xu (Guangzhou University of Chinese Medicine, Guangzhou, China) wrote about the epidemiologic evidence for the impact of COVID-19 vaccination on the risk of Multiple Sclerosis and Ulcerative Colitis occurrence (Shan et al.).

Jia Liu (Wuhan Institute of Virology, Center for Biosafety Mega-Science, Chinese Academy of Sciences, Wuhan, China) introduced a very interesting topic: pathological injury in the tongue and dysgeusis (taste disfunctions) triggered by SARS-CoV-2 infection (Ma et al.).

In the theme of COVID-19 vaccine adverse effects, Diego Dominguez-Balmaseda and co-workers (Faculty of Sports Sciences, Universidad Europea de Madrid, Madrid, Spain) submitted an original research article demonstrating the impact of COVID-19 vaccination in the lower urinary tract (LUT) and overactive bladder (OAB). Interestingly these authors observed gender differences in the vaccination effects. Men vaccinated with Vaxzevria reported a higher number of nighttime voids, while women vaccinated with Spikevax reported more daytime voids (de-la-Plaza-San-Frutos et al.).

Alin Horatiu Nedelcu, Paula Diana Budescu and Elena Jechel (Faculty of Medicine, “Grigore T. Popa” University of Medicine and Pharmacy, Iasi, Romania) presented a review concerning the role of SARS-CoV-2 infection in the development of endocarditis, either directly through active infection or indirectly through a post-infectious immune response. This review also describes a comprehensive approach of how COVID-19 may represent a cornerstone in the analysis of cardiac involvement from viral infections in children and can increase future responsiveness, thereby reducing morbidity from future epidemics/pandemics. (Lupu et al.).

Finally, Hui Zhong, Nan Jiang, Congwen Wei, Wei Chen and Lihua Hou (College of Basic Medical Sciences, School of Medicine, Zhejiang University, Hangzhou, China; Department of Genetic engineering, Beijing Institute of Biotechnology, Beijing, Chin; Department of Pharmacy, Medical Supplies Center of People’s Liberation Army (PLA) General Hospital, Beijing, China) published a very interesting Original Research Article. These authors discovered that SARS-CoV-2 spike protein (SARS-2-S) promote the proliferation and metastasis of hepatocellular carcinoma cells (Huh-7 cells) through exosomes derived from syncytia (Li et al.). It is now very clear that SARS-CoV-2 virus is accountable for a multiple organ dysfunction syndrome, and even COVID-19 vaccines may have adverse effects in rare cases. It was suggested that cancer is a possible secondary effect of SARS-CoV-2 infection, but clinical and experimental studies are still controversial. Thus, a Research Topic solely about potential effects of SARS-Cov-2 and its vaccines on cancer cells are very much needed.

Feedback from Frontiers has been overwhelmingly positive, with all of the papers of this Research Topic having more than 1,000 views and most of them already with citations. We hope you will be informed by this e-book, as well as enjoy the authors’ research contributions, that the presented data and ideas will drive forward the field toward better control or even a cure for COVID-19, and that this Research Topic will promote investigations in SARS-CoV-2 and related syndromes.

